# Editorial: Non-viral vectors for gene therapy/nucleic acid delivery

**DOI:** 10.3389/fbioe.2023.1304769

**Published:** 2023-10-31

**Authors:** Arpita Poddar, Rajkumar Banerjee, Ravi Shukla

**Affiliations:** ^1^ Fiona Elsey Cancer Research Institute, Ballarat, VIC, Australia; ^2^ Ian Potter NanoBiosensing Facility, NanoBiotechnology Research Laboratory (NBRL), School of Science, RMIT University, Melbourne, VIC, Australia; ^3^ Oils, Lipid Science and Technology Division, Indian Institute of Chemical Technology (CSIR), Hyderabad, India

**Keywords:** gene therapy, non-viral vector, nanomaterial, nucleic acid (DNA and RNA), delivery system, nanoparticle, gene editing

In the 19th century, Gregor Mendel identified heritable units, today known as genes, and laid the foundation for an emerging form of treatment known as gene therapy (GT). Subsequently, from the description of double-stranded DNA to completion of the Human Genome Project, GT has become a powerful treatment option for several gene-based diseases. GT involves the intracellular introduction of nucleic acid (NA)-materials for altering host protein expressions in order to cure the diseased state. However, despite nearly 3,000 clinical trials being undertaken (completed or ongoing), GT continues to exist largely only in the experimental phase. A primary challenge holding it back from realising its true potential is the delivery of target gene/NA into cells or tissues (Ginn et al., 2018; Pan et al., 2021). A delivery system, known as a “vector”, is needed to carry such cargo inside the cells. Traditionally, viruses or modified viral-based systems have been used due to high transfection efficacy. However, clinical applications are restricted due to immunogenicity, cytotoxicity, non-targeted insertions, insufficient long-term studies, as well as very high costs.

In this setting, non-viral vectors are emerging with increasing relevance as safer alternatives for bypassing the pathogenicity of viral systems. Lipid-based nanoparticles and cationic polymers represent conventional chemicals that aid with NA-delivery. Such nano/microsystems are the only non-viral vectors in clinical trials but are still impeded by their tendency to aggregate in serum (Pan et al., 2021). On a promising note, successful implementation has recently been highlighted during the unprecedented global efforts to synthesise COVID-19 vaccines. Several of these use lipid nanoparticles to impact the overall immunomodulatory properties of the vaccine itself, in addition to cargo delivery and protection (Guerrini et al., 2022). However, for comparable clinical application in other diseases and therapeutics, the preclinical research stages with similar materials such as liposomes, poly(2-(N,N-dimethylamino)ethyl methacrylate) or poly(l-lysine) are still beset with contradictory results due to varying degrees of aggregation, hemagglutination and low capacities for maintaining DNA integrity and endosomal escape (Poddar et al., 2019a).

Therefore, challenges of transfection efficacy, cargo protection and systemic aggregation are key areas requiring further research-led improvements in this field. However, less than 1% research articles involving delivery systems focus on non-viral options. This slack is picking up, as a plethora of novel strategies, such as unique materials, formulations and innovative applications, are emerging to aid in the advancement towards bench-to-bedside translation (Poddar et al., 2019; Poddar et al., 2020). In this context, this Research Topic brings together several contributions demonstrating the importance of NA delivery utilising non-viral vector-systems (chemicals or particles)—illustrating the potential clinical benefits that can be achieved through GT mediated by safer vector alternatives.

GT modalities are broadly classified into 3 main groups: immunomodulatory, corrective, and cytoreductive or oncolytic approaches. Immunomodulation boosts the host immune system to promote detection and destruction of diseased cells. In such an approach, it is necessary to account for immunomodulation imparted by the delivery system itself. Corrective GT accounts for the bulk of experimental applications with non-viral vectors. It involves replacement or deactivation of target gene expression using materials delivering either targeted or proof-of-concept NAs. For instance, in this Research Topic, diosgenin is reported for the first time to aid in liposome-mediated intracellular NA delivery by facilitating cell membrane permeabilization Lohchania et al. Transfection efficacies differ between delivery of plasmid *versus* mRNA formats. Possible reasons could be differential loading/protection capacities, or varying interactions of the mRNA/protein with the delivery system as compared to DNA. While possessing multiple benefits including safety and ease of optimisations, efficiency continues to be a critical aspect withholding non-viral vectors from reaching the same level of applications as viral vectors, and this study highlights good research carried out in addressing such key issues.

The cytoreductive method, also known as oncolytic or suicide GT, involves delivery of genetic material whose intracellular expression induces cell death. Such utility is illustrated in lung cancer in the next article of the Research Topic. The authors develop a novel suicidal GT system that delivers specific miRNAs and caspase genes leading to apoptosis of non-small-cell-lung cancer (NSCLC) cells *in vitro* as well as in 3D-spheroids Chattopadhyay et al. Such findings pave the way towards increasing therapeutic options for difficult-to-treat diseases with the aid of GT. The following articles in the Research Topic further demonstrate benefits in diseases that are particularly susceptible to GT using a combination of corrective gene-editing paired with other biotechnological methods. Osteogenesis imperfecta (OI) occurs due to abnormal collagen from a mutant COL1A1 gene leading to brittle bones. The authors, in a significant approach, utilise OI patient-derived induced pluripotent stem cells (iPSCs) and transfect them with normal COL1A1 gene through a specialised ‘STAR’ polymer; achieving a remarkable 85% mutation repair efficiency in the iPSCs Fus-Kujawa et al. In the next article, the impact on neurological disorders is evaluated through a well-crafted *in vivo* somatic gene-editing study Neuman et al. Notably, a non-viral novel nanocapsule and a viral delivery system comprising adeno-associated-virus 9 formulations are investigated, with both achieving significant gene editing. Slightly higher efficiency is seen with the viral system. While this difference did not achieve statistical significance, it is consistent with the recurrent theme of general lower efficiency seen with non-viral vectors as compared to viruses.

In summary, the different approaches included in this Research Topic have the unified goal of creating safer, less immunogenic, or non-pathogenic alternatives to virus-based systems as GT carriers. Currently, the common challenge for future research in non-viral vectors appear to be the optimisation of transfection efficiency. While such optimisations are required, it is also prudent to keep in mind that if the efficiency achieved by non-viral systems is sufficient to effect clinical relevance, then increasing efficiency may be unnecessary. Furthermore, a better understanding of the immunomodulatory impact of non-viral vectors, for all GT strategies, is necessary. It is critical to address the major factors in gene delivery, i.e., maintaining cargo integrity; successful cellular entry; endosomal escape and nuclear uptake where required; without inciting unwanted immune responses. Overall, we present the reader with a valuable reference of research findings based on the above factors and provide an overview of the great scope of various non-viral gene-delivery systems in the preclinical setting.
